# Microfluidic-Integrated, Ring-Resonator-Assisted Mach–Zehnder Interferometer (μFRA-MZI) as a Label-Free Nanophotonic Sensor

**DOI:** 10.3390/bios15110741

**Published:** 2025-11-04

**Authors:** Yunju Chang, Ethan Glenn Seutter, Zihao Wang, Jiandi Wan

**Affiliations:** 1 Department of Chemical Engineering, University of California, Davis, CA 95616, USA; imychang@ucdavis.edu (Y.C.); egseutter@ucdavis.edu (E.G.S.); 2 Microsystems Engineering, Rochester Institute of Technology, Rochester, NY 14623, USA; zw4491@rit.edu

**Keywords:** ring resonator, Mach–Zehnder interferometer, microfluidics, photonic biosensor

## Abstract

The ring-assisted Mach–Zehnder interferometer (RA-MZI) has high sensitivity and fast optical response time, and it has been used as a label-free nanophotonic biosensor. Most RA-MZI-based biosensors, however, require chemical modification of the ring surface to immobilize biomolecules that can interact with target molecules for sensing. Here, we report a novel microfluidic-integrated RA-MZI (μFRA-MZI) where a microfluidic channel was fabricated right above the photonic ring resonator. μFRA-MZI allows for direct sample delivery to the RA-MZI without chemical modification of the ring surface and measures shifts in the resonance wavelength induced by the presence of target molecules, enabling label-free detection. In order to optimize the sensitivity of μFRA-MZI, seven devices were fabricated with varied design parameters, including the gap distance between the ring and the bus waveguide (G_ring_), the length of the multi-mode interferometer (L_MMI_), and the length of the directional coupler (L_DC_). Photonic characterization showed that the device with G_ring_ = 1.2 μm, L_MMI_ = 15.5 μm, and L_DC_ = 13.5 μm exhibited the highest extinction ratio (ER) compared to the other six devices, consistent with the simulation-optimized design. Testing with NaCl solutions of varying concentrations yielded a bulk sensitivity of 11.48 nm/refractive index unit (RIU) and an ER of 0.41. With the potential to further improve the device’s sensitivity and the ability to detect samples directly in flow without chemical modifications of the ring resonator, μFRA-MZI will provide a robust and effective approach for label-free biosensing.

## 1. Introduction

Si photonic ring resonators are a class of whispering-gallery resonators that can measure changes in the effective refractive index (RI) within an evanescent field. They have been used widely as label-free photonic sensors [1,2,3,4]. A ring-resonator-based photonic sensor is normally composed of a linear Si waveguide that accesses a micro-ring [5]. When light is introduced to the linear Si waveguide, it propagates along the waveguide and couples into the micro-ring at wavelengths that meet an interference-based resonance condition of $m\lambda =2\pi {rn}_{eff}$ [5,6] where *m* is an integer, *r* is the micro-ring’s radius, and *n_eff_* is the effective RI sampled by the optical mode. Thus, when *n_eff_* changes due to, for example, molecular interactions between the sensing and target molecules on the ring’s surface, a corresponding shift in the resonance wavelengths, ${\Delta \lambda }_{resoant}$, is observed, acting as a photonic sensing signal [7]. Previous studies have demonstrated such label-free biosensing by creating a slot waveguide ring resonator where bovine serum albumin (BSA) and anti-BSA molecular binding events on the sensor’s surface were monitored through the measurement of resonant wavelength shifts [8,9]. More recently, an evanescent-field biosensor with high sensitivity was proposed where engineered dielectric grating was used to achieve a high-Q box-like resonance shape. This device was able to monitor changes in the resonance transmission when Immunoglobulin G (IgG) concentration varied from 1 to 500 pg/mL, exhibiting a high bulk sensitivity of 490.49 nm/RIU [10]. However, a ring-resonant-based sensing mechanism requires the ring resonator to be operated at close to the critical coupling point and is highly sensitive to fabrication flaws and environmental temperature fluctuations, resulting in low signal-to-noise ratios [11,12,13,14].

To overcome the limitations associated with measuring ${\Delta \lambda }_{resonant}$ in ring resonators, a Mach–Zehnder interferometer (MZI) has been used to couple with the ring resonator as a readout unit [15,16]. MZI measures the interference intensity due to phase responses of the ring resonator [17,18]. An MZI contains two arms of waveguides with equal lengths [19]. One arm is side-coupled to the ring as the sensing arm, and the other one acts as a reference arm. When an input signal at the ring resonance wavelength is introduced, the ring resonator induces a phase shift to the signal that travels through the sensing arm, followed by interfering destructively or constructively with the signal that propagates through the reference arm. This leads to periodic interference peaks with minimum or maximum values at the MZI output. When spectra responses are changed from the ring resonator due to effective RI variations, the wavelength of these interference peaks shifts due to phase changes in the sensing arm. Such a wavelength shift is similar to ${\Delta \lambda }_{resonant}$ but does not require the ring resonator to be operated at the critical coupling point and thus has high robustness against fabrication deviations. Importantly, the steep change of interference intensity near resonance significantly improves its sensitivity compared to a ring resonator without MZI [15,18]. The high sensitivity of ring-assisted MZI (RA-MZI) [15,16] and the fast optical response time of ring resonators allow for real-time monitoring of surface functionalization and molecular binding kinetics in many biosensing applications [15,20,21].

**Figure 1:**
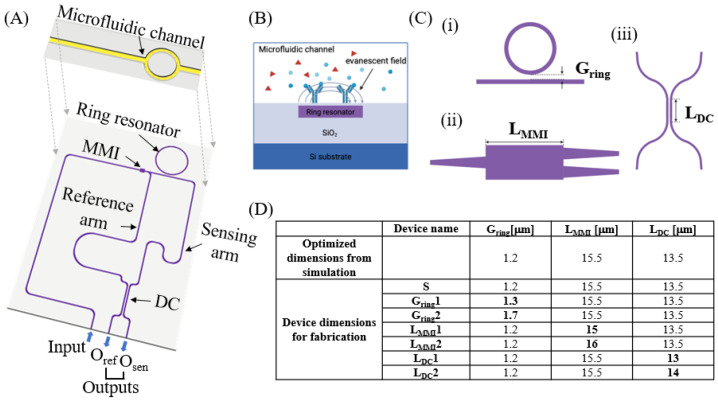
Design of the microfluid–integrated, ring-resonator-assisted Mach–Zehnder interferometer (μFRA-MZI). (**A**) Schematic of the proposed μFRA-MZI. In the RA-MZI (purple color), light from an optical fiber is edge-coupled into the input waveguide and splits into the reference and sensing waveguides using a multi-mode interferometer (MMI). Light from the sensing waveguide is coupled with the ring resonator and combined and affected by light from the reference waveguide in a 2 × 2 directional coupler (DC). Light exits through two output waveguides, i.e., O_ref_ and O_sen_, to an optical fiber for detection. A microfluidic channel (yellow color) is fabricated directly on the top of the ring resonator. (**B**) Schematic of label-free sensing of molecules inside of the microfluidic channel. Red triangles and blue circles/Y shapes indicate biomolecules in the microfluidic channel. (**C**) Design variables for the RA-MZI. (i) Ring gap distance (G_ring_), (ii) MMI length (L_MMI_), and (iii) DC length (L_DC_). (**D**) 7 RA-MZI devices with different design parameters. Optimized dimensions of G_ring_, L_MMI_, and L_DC_ from the simulation were 1.2 μm, 15.5 μm, and 13.5 μm, respectively. Device S had the same dimensions as the optimized dimensions. Devices G_ring_1 and G_ring_2 had varied ring gap distances, L_MMI_1 and L_MMI_2 had varied MMI lengths, and L_DC_1 and L_DC_2 had varied DC lengths, while the other parameters were kept the same as those of device S. The variables that were different from device S are shown in bold.

Most, if not all, ring-resonator-based photonic biosensors, however, require the immobilization of specific sensing molecules on the ring’s surface via multi-step chemical reactions, which normally suffer from low reproducibility [22,23]. More importantly, these chemically modified biosensors can only be used for specific sensing molecules immobilized on the ring’s surface and are not readily implemented in the study of freely interacting (not surface-bound) molecules, exhibiting limited ability to detect different target molecules. Conventional microfluidic-based, albeit not fully integrated, ring resonator systems place the ring resonator inside of a large microfluidic channel (~100 μm), which has a small surface-to-volume ratio and exhibits reduced light–matter interactions (i.e., low sensitivities) [21,22]. Here, we designed, fabricated, and characterized a microfluidic channel integrated directly on top of the ring resonator in a ring-assisted Mach–Zehnder interferometer (μFRA-MZI) (Figure 1A,B). This microfluid–resonator integration facilitates sample delivery through the microfluidic channel and maximizes light–matter interactions within the sensing region, thus enabling sensitive label-free detection of target molecules by measuring RI changes inside of the microfluidic channel [15]. Freely interacting proteins or protein–ligands, for example, can be injected into the microfluidic channel, and RI changes arising from these freely interacting proteins or ligands can be monitored by the RA-MZI to quantify molecular binding kinetics [20,24]. This μFRA-MZI platform thus paves a new route to developing cost-effective, sensitive, and robust RA-MZI photonic biosensors.

## 2. Materials and Methods

### 2.1. Device Fabrication

The μFRA-MZI optofluidic device was fabricated on a 6-inch Si wafer (University Wafer, South Boston, MA, USA). A reticle was produced by Benchmark (Waltham, MA, USA) based on the device’s design. A 4 μm thick layer of silicon dioxide (SiO_2_) as the lower-cladding layer and a 230 nm thick layer of silicon nitride (Si_3_N_4_) were sequentially deposited on the Si wafer using low-pressure chemical vapor deposition (LPCVD) (TYTAN II, Tystar, Garden Grove, CA, USA). The Si_3_N_4_ layer was then patterned using an ASML 5500/300 deep ultraviolet (DUV) stepper (ASML, Celdhoven, The Netherlands) with UV210 positive photoresist and developed using an MF-26A developer (Kayaku Advanced Materials, Westborough, MA, USA). To minimize reflection during exposure, a 0.06 μm thick bottom anti-reflective coating (BARC) was applied beneath the photoresist. After photolithography, the waveguide structures, including the ring resonator and MZI, were selectively etched using inductively coupled plasma reactive ion etching (ICP-RIE) (STS APS, SPTS Technologies, Singapore). Subsequently, another 4 μm thick layer of SiO_2_ was deposited as the upper-cladding layer using LPCVD to encapsulate the waveguides. The microfluidic channel, designed to be 3 μm in width and 3.5 μm in height, was patterned using UV 26 photoresist (2.2 μm-thick) and etched onto the SiO_2_ upper-cladding layer directly on the top of the ring resonator’s structure. An approximately 0.5 μm thick layer of SiO_2_ was intentionally left intact above the ring resonator to prevent damage to it. A facet was patterned using a Karl Suss MA6 mask aligner (SUSS MicroTec SE, Garching, Germany). Facet etching was conducted in two steps, SiO_2_ etching and Si etching. The facet etching extended from the SiO_2_ upper-cladding layer down to approximately 150 μm through the Si substrate. An 8 μm thick AZ 12XT positive photoresist and an AZ MIF 300 developer were used in the deep etching. Deep Si etching was performed using the Bosch process in a Versaline ICP etch system (PlasmaTherm, Saint Petersburg, FL, USA). The microfluidic channel was sealed using polydimethylsiloxane (PDMS), which was prepared by mixing a prepolymer and a curing agent at a 10:1 weight ratio (Sylgard 184 (Dow Chemical, Midland, MI, USA)). The mixture was thoroughly stirred to ensure uniform dispersion and then degassed in a vacuum chamber for 1 h to remove air bubbles. Then, PDMS was poured over a microfluidic master (50 μm in width, 40 μm in height, and with some length) in a Petri dish and cured at 70 °C for 2 h. After curing, the PDMS was cut into small pieces, and inlet and outlet holes were punched using a 1 mm biopsy punch (BP-10F, Kai Medical). PDMS and a photonic chip were treated with oxygen plasma and sealed together at 80 °C for 1 h. The size of a single device was 1.7 cm × 2.5 cm × 0.5 cm. Note that the eight squares near the center of the device were for electrical contact pads, which were not utilized in this project.

### 2.2. SEM Imaging

Prior to imaging, sputter coater (Q150RES, Quorum, San Jose, CA, USA) was used to coat 5 nm thick gold/palladium (60/40) film to prevent the charging effect. Images of the ring resonator, microfluidic channel, multimode interferometer (MMI), and directional coupler (DC) were taken using a Field Emission Scanning Electron Microscope (FE SEM) (Nova Nano SEM 430, FEI, Hillsboro, OR, USA). SEM imaging was performed at an acceleration voltage of 10 kV with a working distance of 5 mm. To examine the cross-sectional alignment between the ring resonator and the microfluidic channel, a Focused Ion Beam Scanning Electron Microscope (FIB-SEM) (Scios Dual Beam FIB/SEM, FEI, Hillsboro, OR, USA) was used to mill a small section of the microfluidic channel, exposing the underlying ring resonator. A gallium (Ga^+^) ion beam was operated at 0.5 nA for initial bulk milling, followed by 0.2 nA for fine polishing to achieve a clean cross-section. Imaging was then conducted using the secondary electron (SE) detector at an acceleration voltage of 5 kV and a working distance of 7 mm.

### 2.3. Fiber Array and Photonic Chip Alignment

Fiber array (single mode, OZ optics) was secured in a fiber array holder (HFA001, Thorlabs, Newton, NJ, USA) that was controlled by a 6-axis NanoMax Stage (MAX601D, Thorlabs, Newton, NJ, USA). The fabricated photonic chip was placed on a vacuum waveguide holder (HWV001, Thorlabs, Newton, NJ, USA) controlled by a 3-axis MicroBlock Compact Flexure Stage (MBT616D, Thorlabs, Newton, NJ, USA). A visual fault locator (30KM VFL, VFLTOOL, Houston, TX, USA) with a wavelength of 650 nm and a digital microscope (Jiusion-1000×, Jiusion, Shenzhen, China) were used to visualize the alignment.

### 2.4. Photonic Characterization Setup

A tunable laser (Velocity TLB-6728, Newport, Newport Beach, CA, USA) was used to sweep the wavelength from 1530 nm to 1570 nm with 0.1 nm intervals to identify the resonance wavelengths. An infrared camera (SU320KTSW-1.7RT, Goodrich, Charlotte, NC, USA) was utilized to capture the trapped light inside of the ring resonator at a resonance wavelength. After propagating through the ring resonator and the MZI structure, the output light from both outlets, O_ref_ and O_sen_, was collected by the balanced optical receiver (Model 2017, Newport, Newport Beach, CA, USA). To test the sensitivity of the device, NaCl solution was prepared by dissolving NaCl powder (Sigma-Aldrich, St. Louis, MO, USA) in deionized water with concentrations ranging from 5% to 15% weight/weight. The NaCl solution was introduced into the microfluidic channel via a syringe pump (Pump 11 Pico Plus Elite, Harvard Apparatus, Holliston, MA, USA) at 0.05 mL/min. Once the microfluidic channel was filled with the solution, we allowed 5 min for the output optical signals to be stabilized. Then, we swept the wavelength around the resonance wavelength to measure the extent of the resonance wavelength shift corresponding to the change in NaCl solution. RI values of NaCl solutions in the infrared region were obtained using the third-order polynomial fit provided by Saunders et al. [25], with the weight percentage of NaCl as the independent variable. After measurement, the microfluidic channel was washed thoroughly with deionized water before proceeding to the next NaCl solution with a different NaCl concentration. This process was repeated three times for each NaCl solution concentration.

## 3. Results and Discussion

### 3.1. Design Principles

The μFRA-MZI optofluidic device shown in Figure 1A is composed of a ring resonator, an MZI, and a microfluidic channel. The microfluidic channel is designed to sit directly on top of the ring resonator such that any RI changs inside of the microfluidic channel can be captured by the ring resonator underneath. This approach is particularly advantageous to measure RI changes resulting from freely interacting (not surface-bound) proteins or protein–ligand interactions in flow conditions, such as the mechanoenzymatic cleavage of the von Willebrand Factor in circulation [26]. Conventional RA-MZI or ring-only sensors require chemical functionalization of the device to immobilize sensing molecules for detection and thus have limited capability to monitor RI changes from freely interacting proteins.

In the μFRA-MZI optofluidic device, light is coupled into the waveguide from the input waveguide and propagates to a 1 × 2 multimode interferometer (MMI) [17,27]. In the MMI, light splits into two equivalent beams and propagates to the reference and sensing arms of the MZI, respectively [17,19]. In the sensing arm, light couples into the ring resonator through the evanescent field at resonance wavelengths (Figure 1B) [4,5]. Because the ring resonator is located right below the microfluidic channel, RI changes inside of the microfluidic channel will trigger a corresponding shift in the resonance wavelength [15]. Light in the reference and sensing arms will then interfere via the directional coupler (DC) [16] and exit through two output waveguides, i.e., O_ref_ and O_sen_, to the optical fiber array for detection. Note that the distance between the surface of the ring resonator and the bottom of the microfluidic channel is ~0.5 μm. Such a short distance ensures the penetration of the evanescent field from the ring to the microfluidic channel. The width of the microfluidic channel, i.e., ~3 μm, is larger than the width of the ring, so most of the evanescent field will be within the channel. This microfluid–resonator integration thus enables sufficient light–matter interactions inside of the microfluidic channel for sensitive RI detection.

In designing the RA-MZI, we considered three key parameters. (i) The gap distance between the ring resonator and the bus waveguide (G_ring_). G_ring_ determines the coupling regime between the ring resonator and the bus waveguide [11], and thus is important to obtain a high signal-to-noise ratio. (ii) The length of MMI (L_MMI_), which controls the optical power splitting of the incoming light [17,27]. (iii) The coupling length of DC (L_DC_), which regulates the interference pattern of the light from the reference and sensing arms, ensuring the cancelation of common mode noise [28] to increase the signal-to-noise ratio. Here, we optimized G_ring_, L_MMI_, and L_DC_ through simulation and fabricated and tested seven devices experimentally using different G_ring_, L_MMI_, and L_DC_ (Figure 1C). Device S had the simulation-optimized G_ring_, L_MMI_, and L_DC_. The other six devices were designed with varied G_ring_ (devices G_ring_1 and G_ring_2), L_MMI_ (devices L_MMI_1 and L_MMI_2), and L_DC_ (devices L_DC_ 1 and L_DC_2), while other parameters were kept the same as those of device S.

### 3.2. Fabrication and Dimensional Characterization

**Figure 2:**
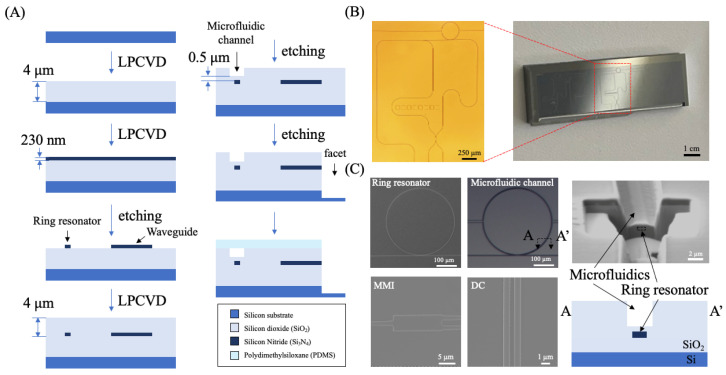
Fabrication process of the μFRA-MZI. (**A**) Flow chart of the fabrication process. Silicon dioxide (SiO_2_) and silicon nitride (Si_3_N_4_) were deposited using low-pressure chemical vapor deposition (LPCVD) sequentially on a Si wafer as the bottom cladding and the waveguide, respectively. The waveguide structure and the ring resonator were patterned by etching Si_3_N_4_ using reactive ion etching (RIE), followed by SiO_2_ deposition via LPCVD as the top cladding. The microfluidic channel and facet were etched by RIE on the top SiO_2_ cladding and sealed using polydimethylsiloxane (PDMS). Note that there was a 0.7 μm thick layer of SiO_2_ between the ring resonator and the bottom of the microfluidic channel to protect the ring resonator’s surface without compromising its sensitivity. (**B**) Representative bright-field images of a fabricated μFRA-MZI device (i.e., L_DC_1). The size of the entire device was 1.7 cm (width) × 2.5 cm (length) × 0.5 cm (height). Eight square electrical contact pads in the middle of the device were not fabricated or used in this project. (**C**) Representative Scanning Electron Microscope (SEM) images of the ring resonator, microfluidic channel, MMI, and DC. The cross-sectional image (right) confirmed the alignment of the microfluidic channel with the ring resonator. The width and height of the microfluidic channel were 3 μm and 3.4 μm, respectively.

**Figure A1:**
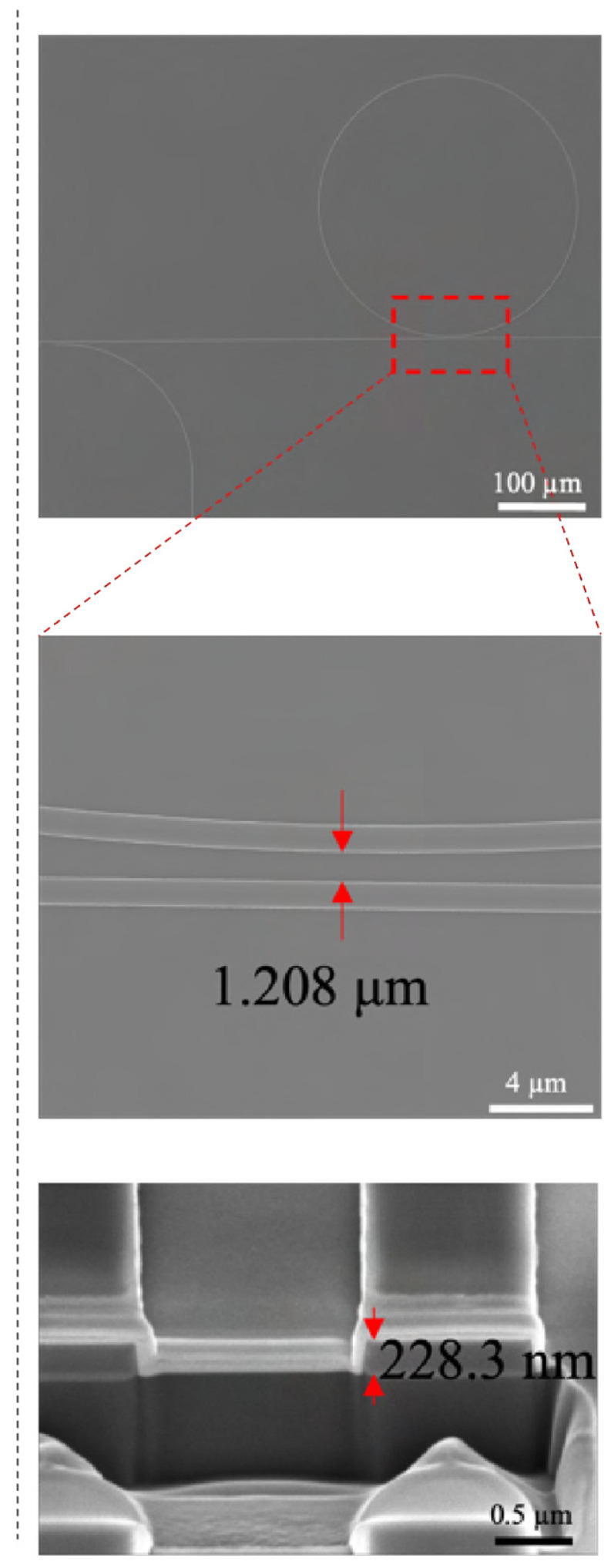
SEM images ring resonator. The overview (**top**) and zoomed-in images (**middle**) of each structure are shown. Additionally, the FIB-SEM image (**bottom**) shows the cross-section of the gap between the ring resonator and the bus waveguide and between the directional coupler. Red arrows indicate the distance measured.

**Figure A2:**
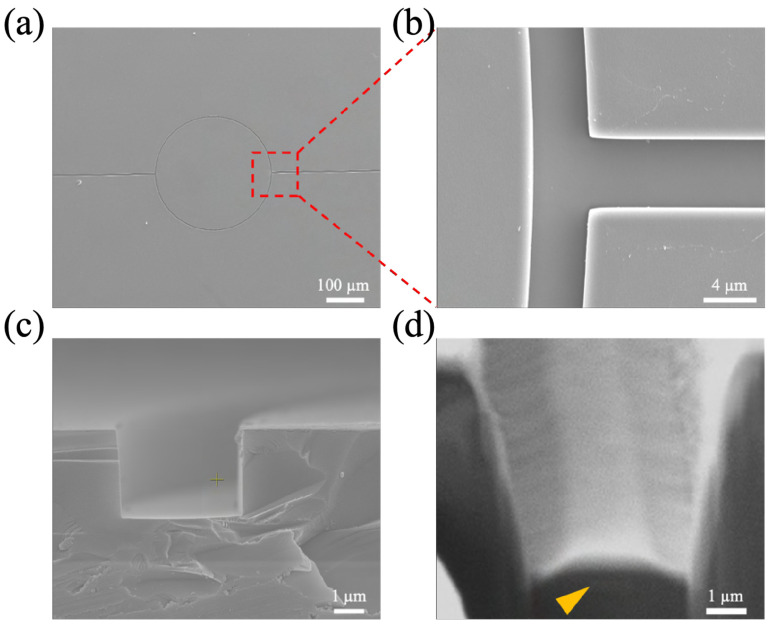
SEM images of microfluidic channel. Top (**a**,**b**) and cross-sectional views of the microfluidic channel (**c**). (**d**) The cross-sectional view of the FIB-SEM image shows that the microfluidic channel is in alignment with the ring resonator (yellow arrow).

The fabrication process involved sequential deposition of dielectric layers, photolithography, etching of optical and fluidic structures, and, finally, sealing with PDMS (Figure 2A) [29]. Note that deep Si etching was conducted using the Bosch process [30], which alternated passivation and etching cycles and produced smooth and vertical facets for efficient optical fiber coupling. This step exposed the inlet and outlets of the waveguide, facilitating light coupling between waveguides and the optical fibers. Representative bright-field images of a fabricated device are presented in Figure 2B, where an optical microscope image of a single device is shown on the left and a diced chip with five devices is shown on the right. A single device was approximately 1.7 cm in width and 2.5 cm in length. Figure 2C shows the SEM images of the ring, MMI, and DC, confirming that the fabrication of the waveguide structure was consistent with design dimensions with variations between 10 and 100 nm (Figure A1). A cross-sectional FIB-SEM image of the microfluidic channel (Figure A2) demonstrates the formation of nearly vertical side walls (Figure 2C). The depth of the microfluidic channel measured from FIB-SEM is 3.4 µm, close to the designed depth of 3.5 µm. The width of the channel is 3 μm. The bottom of the channel was located 0.7 µm above the ring resonator. The results indicate a well-controlled etching process and precise alignment between the microfluidic channel and the ring resonator, ensuring that the fluidic region is positioned correctly on the top of the ring resonator.

### 3.3. Optical Characterization and Extinction Ratio Analysis

**Figure 3:**
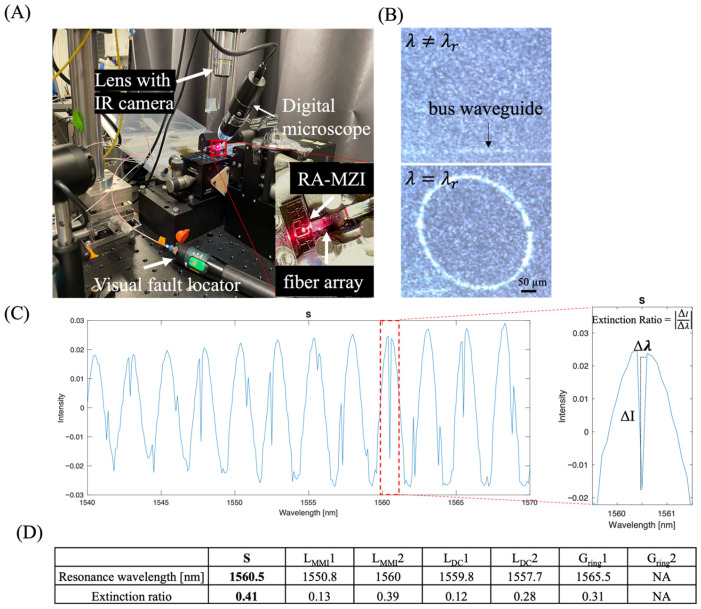
Determine the extinction ratio (ER) of RA-MZI fabricated with different design parameters. (**A**) Representative bright-field image of the experimental setup. A 650 nm red light was used to visualize and facilitate light–waveguide coupling. For precise alignment between the RA-MZI device and the fiber array, a visual fault locator, a digital microscope, and an IR camera were employed. (**B**) Representative infrared images showing the coupling between the bus waveguide and the ring resonator when a resonance wavelength ($\lambda_{r}$) was achieved. (**C**) Typical data plot used to calculate the ER of an RA-MZI. ER was defined as the difference in the output power intensity (∆I) divided by the difference in the wavelength (∆λ). Data were obtained using device S. (**D**) Experimentally measured maximum ER in fabricated devices. Device S showed the highest maximum ER of 0.41 at the resonance wavelength of 1560.5 nm among all devices. In the case of G_ring_2, the gap distance was too large, preventing light from coupling into the ring resonator, resulting in no observable resonance peaks.

The μFRA-MZI devices were optically characterized using the setup shown in Figure 3A. We aligned a single-mode optical fiber array with the RA-MZI using a red laser (650 nm), a visual fault locator, and a digital microscope. After alignment, a tunable light source with a wavelength ranging from 1530 nm to 1570 nm was coupled into the input of the waveguide via the single-mode fiber array. When the laser wavelength matched the ring’s resonance condition, light confinement in the ring became visible in an infrared camera (Figure 3B). This confirmed the precise alignment prior to full optical characterization. After light propagated through the ring resonator and MZI structure, the output light from both outlets was collected by a balanced optical receiver. This receiver converts the difference in light intensity between the two outputs into a voltage signal. The voltage signals were recorded for each wavelength by an oscilloscope and plotted as the transmission spectrum.

**Figure A3:**
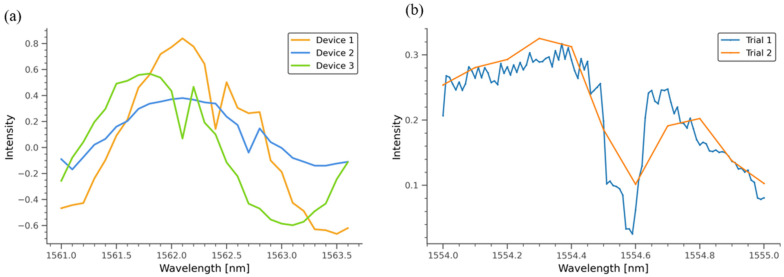
Optical characterizations to examine the reproducibility of the device’s fabrication. (**a**) Resonance peaks in three independently fabricated devices with the same configuration (device S) were 1562.1, 1562.4, and 1562.7 nm. (**b**). Repeated measurements in device S showing a constant resonant peak at 1554.6 nm.

A typical transmission spectrum exhibited positive, broad bandwidth peaks along with negative, sharp bandwidth peaks (Figure 3C). These positive, broad bandwidth peaks were primarily due to light interference, whereas negative, narrow bandwidth peaks represented resonance peaks [5,6]. Note that these resonance peaks were reproducible between independently fabricated devices with the same configuration (device S) (Figure A3). For each individual device, the resonant peak remained constant over repeated measurements, confirming the stability of the device’s response and validating that any spectral shift arises only from changes in the surrounding optical environment. The ratio of the transmission intensity (∆I) vs. wavelength (∆λ) of the resonance peak, namely, the extinction ratio (ER = ∆I/∆λ), is highly sensitive to changes in effective RI [2,7], allowing for precise measurement of small wavelength shifts. Figure 3C illustrates how ER was defined at a resonance wavelength in the transmission spectrum of device S. A high ER enables the sensor to detect small changes in the concentration of an analyte, which, in turn, effectively lowers the limit of detection.

The maximum ERs measured for different devices are shown in Figure 3D. Among the seven fabricated devices, device S showed the highest ER of 0.41 at a resonance wavelength of 1560.5 nm, which was approximately 7.3 dB and close to 10–15 dB in most silicon ring resonators [31]. The decrease in L_MMI_ by 0.5 µm (from 15.5 µm in device S to 15 µm in device L_MMI_1) resulted in a ~70% reduction in ER (ER = 0.41 and 0.13 for device S and device L_MMI_1, respectively). The increase in L_MMI_ by 0.5 µm (from 15.5 µm in device S to 16 µm in device L_MMI_2) led to a ~5% reduction in ER (ER = 0.41 and 0.39 for device S and device L_MMI_2, respectively). Because the MMI in device S was expected to have a balanced power splitting ratio (i.e., 50:50), a decrease in the length of MMI significantly affects the power splitting ratio, resulting in degraded ER.

Meanwhile, an increase or decrease in the coupling length (L_DC_) relative to the L_DC_ in device S (L_DC_ = 13.5) resulted in a decrease in ER in devices L_DC_1 and L_DC_2 (ER = 0.12 and 0.28 for L_DC_1 and L_DC_2, respectively). Such a reduction in ER could be due to insufficient common mode cancellation via the balanced detector [28]. Insufficient cancellation of the destructive interference in the DC results in shallower dips in the transmission spectrum and lower ERs [15,17,28]. Lastly, an increase in the gap distance between the ring and the bus waveguide (G_ring_) from 1.2 µm to 1.3 µm led to a decrease in ER from 0.41 to 0.31 in device S and G_ring_1, respectively. This was expected, as the coupling transitions from critical coupling to over-coupling regimes [5]. As the gap distance further increased to 1.7 µm in G_ring_2, no resonance peak was identified.

### 3.4. Evaluation of Optical Sensitivity of the µFRA-MZI

**Figure 4:**
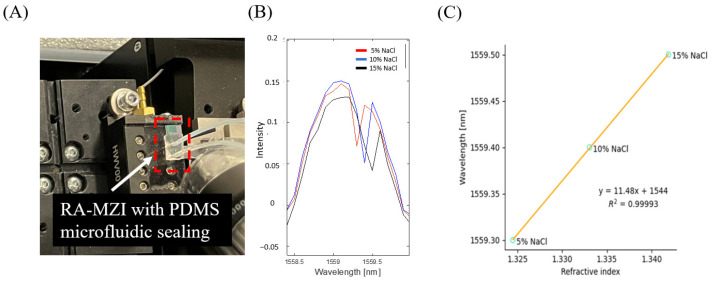
Determination of the optical sensitivity of optimized μFRA-MZI (device S). (**A**) Experimental setup with fluid flow in microfluidics. Device S with PDMS microfluidic sealing was placed on the waveguide holder. Different concentrations of NaCl were introduced to the microfluidic channel via a syringe pump. (**B**) The resonance wavelength increased with the increase in NaCl concentration. (**C**) Wavelength shift as a function of bulk RI change. Sensitivity was calculated as the slope of the fitting curve, which is 11.48 nm/RIU.

We next examined the sensitivity of device S, as it had the highest ER among the seven fabricated μFRA-MZI devices. To do so, various concentrations of sodium chloride (NaCl) (5%, 10%, and 15% by weight) solutions were introduced into the microfluidic channel, as shown in Figure 4A. The device was placed on the waveguide holder, and the NaCl solutions were introduced via a syringe pump, ensuring a controlled flow rate over the sensing region. As the concentration of NaCl solution increased from 5% to 15%, the resonance wavelength shifted from 1559.3 nm to 1559.5 nm (Figure 4B). The RI of NaCl solutions in the infrared region is 1.324, 1.333, and 1.342 for 5%, 10%, and 15% NaCl, respectively (calculated using the polynomial reported by [25]). We plot the resonance wavelength vs. RI at different NaCl concentrations in Figure 4C, where a linear fit provides a slope representing the sensitivity (S = ∆λ/∆n) of device S.

The calculated sensitivity in device S was 11.48 nm/RIU, which was low compared to other RA-MZI sensors [15,16] and ring/MZI/Vernier sensors [7,18,20,21,32]. We speculated that the low sensitivity is partially due to the thermal drift induced by flowing fluids through the microfluidic channel, which may cause changes in RI in the sensing channel [14,33,34,35]. Furthermore, silicon nitride has a relatively high thermo-optic coefficient [14,33,34,35], which can lead to large RI changes as the temperature varies. Johnson et al. have shown variations in RI due to temperature change, and the coefficients of thermal-induced RI change were ~2.45 × 10^−5^ RIU/°C and ~0.95 × 10^−5^ RIU/°C for silicon nitride and silicon dioxide, respectively [33]. Such thermal-induced RI variations in RA-MZI sensors had a significant impact on resonant peak shifts [36] and thus could affect negatively the device’s sensitivity. Future work with integrated heaters to minimize temperature variations in the RA-MZI sensor to effectively maintain a constant temperature will enhance the sensitivity of the optical measurements.

## 4. Conclusions

This work presented the design, fabrication, and characterization of an RA-MZI photonic sensor with an integrated microfluidic channel directly above the ring resonator. We investigated the effects of key design parameters on the ER of the fabricated devices and showed that the optimized device exhibited the highest ER of 0.41 and a bulk sensitivity of 11.48 nm/RIU. This microfluidic-integrated RA-MZI photonic sensor facilitates sample delivery through the microfluidic channel and enables RI detection without chemical functionalization of the device, complementing conventional RA-MZI or ring-only sensors that require chemical functionalization for sensing. Note that the present μFRA-MZI does not exclude chemical functionalization for sensing, and the microfluidic channel can be chemically modified for sensing if needed. Therefore, μFRA-MZI has potential for multifaceted applications in a wide range of fields, including, but not limited to, point-of-care diagnostics, environmental sensing, and fundamental studies in protein interaction dynamics. Meanwhile, we noticed that the bulk sensitivity was lower than those reported in other RA-MZI sensors in the literature. These differences likely arise from environmental temperature fluctuations during measurement. By adding a temperature control apparatus to μFRA-MZI, we expect that the sensitivity of μFRA-MZI will be improved significantly, further expanding its applications in fields related to photonic biosensing and biosensors.

## Data Availability

The data supporting this article have been included as part of the Appendix A.

## Abbreviations

BARC	Bottom Anti-Reflective Coating
DC	Directional Coupler
DUV	Deep Ultraviolet
ER	Extinction Ratio
FE-SEM	Field Emission Scanning Electron Microscope
FIB-SEM	Focused Ion Beam–Scanning Electron Microscope
FRA-MZI	Microfluidic-Integrated, Ring-Resonator-Assisted Mach–Zehnder Interferometer
Ga^+^	Gallium Ion
Gring	Ring–Bus Waveguide Gap Distance
ICP-RIE	Inductively Coupled Plasma Reactive Ion Etching
LDC	Directional Coupler Length
LMMI	Multimode Interferometer Length
LPCVD	Low-Pressure Chemical Vapor Deposition
MMI	Multimode Interferometer
MZI	Mach–Zehnder Interferometer
NaCl	Sodium Chloride
Oref	Reference Output Port
Osen	Sensing Output Port
PDMS	Polydimethylsiloxane
RA-MZI	Ring-Assisted Mach–Zehnder Interferometer
RI	Refractive Index
RIU	Refractive Index Unit
SE	Secondary Electron
Si_3_N_4_	Silicon Nitride
SiO_2_	Silicon Dioxide
UV	Ultraviolet
VFL	Visual Fault Locator
µFRA-MZI	Microfluidic-Integrated RA-MZI (same as FRA-MZI)

## Appendix A

Figure A1 (SEM of ring resonator and gap, including FIB-SEM of Gring); Figure A2 (SEM of microfluidic channel, top and cross-section views); Figure A3 (reproducibility tests).
